# Alteration of fimbria-mediated biofilm formation and virulence in the zoonotic pathogen *Edwardsiella piscicida* by sub-inhibitory concentrations of erythromycin exposures

**DOI:** 10.1128/spectrum.00366-26

**Published:** 2026-04-27

**Authors:** Jeong Woo Park, Se-Won Baek, Ho Sung Kim, Aaron M. Yerke, Yogini S. Jaiswal, Leonard L. Williams, Hyejung Jung, Jin-Young Yang, Ho Young Kang, Sungmin Hwang, Ki Hwan Moon

**Affiliations:** 1 Department of Convergence Interdisciplinary Education of Maritime & Ocean Logistics, Korea Maritime & Ocean University 34969 , Busan, Republic of Korea; 2 Division of Convergence on Marine Science, Korea Maritime & Ocean University 34969 , Busan, Republic of Korea; 3 Department of Bioinformatics and Genomics, University of North Carolina at Charlotte 14727, Charlotte, North Carolina, USA; 4 Center for Excellence in Post Harvest Technologies, North Carolina Agricultural and Technical State University 536365, Kannapolis, North Carolina, USA; 5 Biotechnology Research Division, National Institute of Fisheries Science, Busan, Republic of Korea; 6 Department of Biological Sciences, Pusan National University 124655, Busan, Republic of Korea; 7 Department of Microbiology, Pusan National University, Busan, Republic of Korea; Texas A&M University, College Station, Texas, USA

**Keywords:** sub-inhibitory concentration, erythromycin, fimbriae, biofilm, *Edwardsiella piscicida*

## Abstract

With a global rise in industrialization and chemical processing units, the introduction of anthropogenic pollutants into the environment has been on a rise. Aquatic pollutants, unlike terrestrial pollutants, are easily diluted in water environments and exist at a sub-inhibitory concentration (sub-IC). At sub-IC, they do not directly inhibit bacterial growth but can modulate gene expression profiles. Aquaculture industry relies heavily on the use of antibiotics for control of fish pathogens, and the indiscriminate use of antibiotic agents increases antibiotic resistance in the aquatic environment. Previous studies have reported that sub-IC of antibiotics are able to regulate biofilm formation in a variety of pathogenic bacteria. To understand the environmental signals in the biofilm formation of *E. piscicida*, we hypothesized that the biofilm of *E. piscicida* can be regulated by sub-IC of erythromycin, which is widely used antibiotic in aquaculture. Our results indicate that at sub-IC of erythromycin, biofilm formation was induced due to increased type 1 fimbrial expression. This caused an increased colonization of the host and hyper-virulence in *E. piscicida*. Our study reveals a new aspect of antibiotic functioning as a signaling molecule that modulates biofilm-related-gene expression and virulence in fish pathogen. This study demonstrates the comprehensive molecular response of the fish pathogen to environmentally discharged erythromycin. Additionally, the study provides scientific evidence for the hypertoxicity of pathogens through the indiscriminate use of antibiotics—ultimately leading to detrimental effects to the fishery industry.

**IMPORTANCE:**

Antibiotics released into aquatic environments often persist at sub-inhibitory concentrations, where they no longer suppress bacterial growth but instead act as signaling molecules. Here, we show that sub-inhibitory erythromycin enhances biofilm formation and virulence in the fish pathogen *Edwardsiella piscicida* by upregulating type 1 fimbriae. This response promotes host colonization and hypervirulence, demonstrating that environmentally relevant antibiotic exposure can unintentionally increase pathogenic potential. Our findings provide *in vivo* evidence that sub-therapeutic antibiotics reshape bacterial behavior and host-pathogen interactions. This study highlights an underappreciated ecological and economic risk of indiscriminate antibiotic use in aquaculture, with direct implications for fish health, disease management, and environmental safety.

## INTRODUCTION

With increase in the global demand for aquaculture products, the aquaculture industry has seen a significant growth (1). To meet ever-increasing demands and be able to supply healthy and fresh products, the aquaculture industry has indiscriminately adopted the use of antibiotics. Use of overcrowded fish raising spaces to meet the demands for supply has led to a high outbreak of aquatic diseases. Bacterial pathogens can spread rapidly in limited spaces and cause infections and mortality in raised fish, ultimately leading to significant economic and environmental losses (2). Some of the pathogenic microorganisms causing these bacterial diseases include *Aeromonas hydrophila*, *Streptococcus iniae*, *Vibrio* spp., and *Edwardsiella* spp. (3). Out of all the fish pathogens, *Edwardsiella* spp. is known to cause the most serious damaging effects (2–5).

*Edwardsiella* consists of five genera: *Edwardsiella ictaluri*, *Edwardsiella hoshinae*, *Edwardsiella tarda*, *Edwardsiella anguillarum*, and *Edwardsiella piscicida* (6)*. E. piscicida* was identified in 2012 and is a conditionally aerobic Gram-negative bacterium belonging to the Enterobacteriaceae (7). *E. piscicida* is known as a fish-borne bacterium that causes diseases in fish in seawater and freshwater and is a zoonotic infection strain that causes diseases in humans (7–9). A representative disease is edwardsiellosis, which causes fatal diseases in aquaculture fish including flounder, turbot, channel catfish, tilapia, red sea bream, and Japanese eel (10–14). *E. piscicida* is known to be more pathogenic than other strains in aquaculture farms, causing huge losses in Asia and the United States where aquaculture is developed. Edwardsiellosis symptoms include septicemia, peritonitis, petechial hemorrhage, and abscess in fish. In humans, it is known to cause gastroenteritis and meningitis (5, 15–17). In recent years, several studies on *Edwardsiella* spp. virulence factors have been conducted (18). Representative virulence factors include type III secretion system (T3SS), type VI secretion system (T6SS), hemolysins, flagellin, serum resistance, and biofilm formation. Among these known virulence factors, biofilm is widely recognized as one of the major contributors to bacterial pathogenesis and antibiotic resistance in many pathogenic microorganisms (19).

Biofilm formation is regulated by a complex network of cellular factors and environmental cues, including the secretion of exopolysaccharides, outer membrane proteins, salt concentration, quorum-sensing signals, and surface appendages such as fimbriae (20–22). In this study, we investigated how sub-inhibitory concentrations (sub-IC) of erythromycin influence biofilm development, with a particular focus on the role of type 1 fimbria expression. Type 1 fimbriae are hair-like bacterial appendages which are composed primarily of the structural protein FimA and are topped by the lectin-like protein FimH (23–25). These fimbriae are a virulence factor of pathogenic microorganisms that enable adhesion, colonization, and infection in host cells (26–28).

To reduce economic damages to production due to infections, many antibiotics are used in aquaculture (29, 30). In aquaculture, erythromycin is widely used as a therapeutic antibiotic against bacterial infections and, indeed, macrolides antibiotics have relatively high residual levels in aquatic system unlike β-lactam antibiotics that are hydrolyzed (31). Moreover, antibiotic residues released into aquatic environments can disperse rapidly and become diluted to sub-IC, where they may not suppress bacterial growth but can influence pathogen growth. *E. piscicida*, a major fish pathogen, employs type 1 fimbriae to mediate biofilm formation, a key virulence factor that enhances persistence and host colonization. Recent studies have shown an increase in biofilm formation in pathogenic microorganisms exposed to sub-IC antimicrobial compounds (32–35). For instance, Lima-e-Silva et al. showed that sub-inhibitory concentrations of rifampicin strongly stimulated biofilm production in *S. aureus* (36). Sub-IC of antibiotics can alter bacterial physiology, including the regulation of fimbrial expression and biofilm formation in several bacterial pathogens (37–41). In aquaculture environments, where residual antibiotics are commonly detected, bacteria are frequently exposed to such sub-IC levels. Despite this ecological relevance, the effects of sub-IC antibiotics on fimbria-mediated biofilm development and subsequent pathogenicity remain insufficiently characterized. This gap in knowledge is especially pronounced for aquatic pathogens such as *Edwardsiella piscicida*.

Here, we evaluated the effect of the sub-IC of erythromycin on type 1 fimbria-mediated biofilm formation in *E. piscicida* and assessed the resulting alterations in virulence using zebrafish infection models. To address both cellular and host-level consequences, we further examined bacterial adhesion and invasion in cell lines and determined how these changes translate into *in vivo* pathogenic outcomes in zebrafish hosts.

## RESULTS

### Determination of the sub-inhibitory antimicrobial concentration for erythromycin

**Fig 1:**
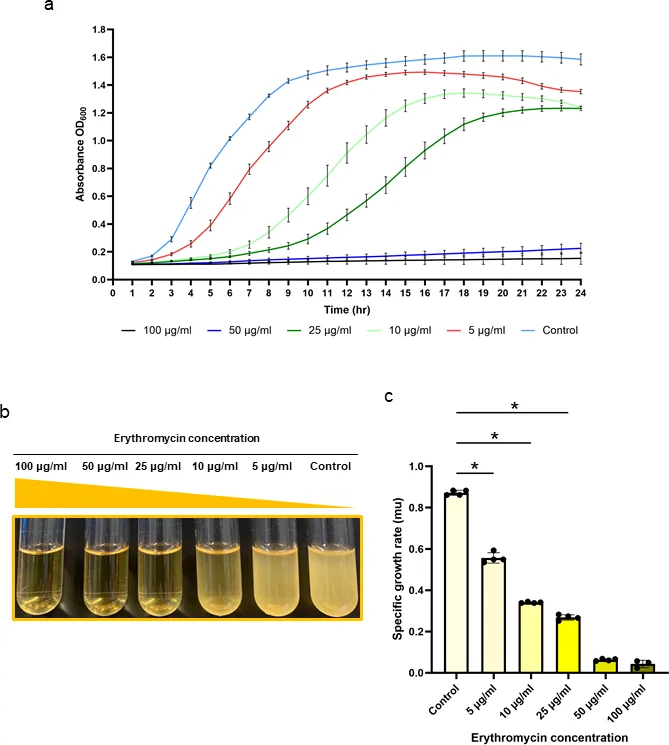
Determination of growth kinetics of *E. piscicida* under gradient concentrations of erythromycin. *E. piscicida* was grown in Brain Heart Infusion (BHI) broth with or without erythromycin. (**a**) The 96-well microplate was incubated in a plate reader with double orbital shaking at 27°C. Bacterial growth was detected at an absorbance of 600 nm and read at intervals of 1 h. (**b**) Identification of the sub-inhibitory concentration (sub-IC) for erythromycin. Cells were grown in 3 mL of BHI medium with the indicated percentages of erythromycin. (**c**) Comparison of specific growth rates at the exponential (7 h) and stationary (17 h) phase. Values are the means ± standard deviations (error bars) from three independent experiments, with three technical replicates in each experiment. Asterisks represent significant differences (*P* < 0.0001) between the growth conditions as determined by *t*-tests.

Erythromycin is one of the widely used antibiotics in aquaculture (42). Since chemicals including antibiotics can be rapidly diluted to a lower concentration after introduction into the aquatic environment, erythromycin, which is a range of 5 to 100 µg/mL, was tested on fish pathogen *E. piscicida* to observe the effect of a series of concentrations of erythromycin. While wild-type without erythromycin reached a stationary phase within 10 h after inoculation, longer time (13, 17, and 22 h) was taken as the concentration of erythromycin increased until optical density (OD_600 nm_) maximized (5, 10, and 25 µg/mL, respectively) (Fig. 1a). Cells incubated with erythromycin concentrations greater than 50 µg/mL did not survive (Fig. 1a and b). A viable counting assay was performed to measure the number of live cells as an optical density-based method is doubted to occur false positive counts (43). At the exponential phase, a 50% reduction in the number of cells (2.6 × 10^8^ CFU) was found with the erythromycin treatment of 5 µg/mL, compared to the untreated (5.1 × 10^8^ CFU). At the stationary phase, there was no significant difference in the CFU between cells with or without 5 µg/mL of erythromycin treatment (Fig. 1c). Therefore, 5 µg/mL of erythromycin was selected as a subinhibitory concentration (sub-IC) where the cell growth was retarded, but cell density recovered as much as the cell group without the erythromycin treatment. Though the number of cells was similar in the stationary phase regardless of the amount of erythromycin, the specific growth rate was gradually reduced as increased the concentration of erythromycin (from 5 to 25 µg/mL, Fig. 1).

### Transcriptional profiling at sub-IC of erythromycin

**Fig 2:**
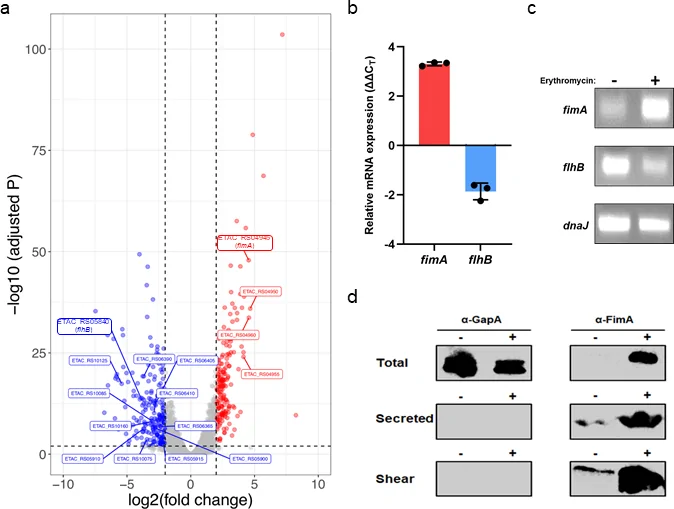
Gene expression changes in *E. piscicida* with sub-IC of erythromycin by *in silico* and *in vitro* analyses. The alteration of gene expression levels of *E. piscicida* when exposed to sub-IC of erythromycin were determined by RNA-seq, qRT-PCR, and immunoblot analyses. (**a**) Determination of the differentially expressed genes (DEGs) from the contrast of erythromycin and non-treated samples. Each gene analyzed was visualized as a dot in the volcano plot. The genes that are upregulated and downregulated were colored in red and blue, respectively. Genes involved in Type 1 fimbrial formation or in flagellar assembly and chemotaxis were selected to assess their expression levels. (**b**) qRT-PCR analysis was performed to estimate gene expression about *fimA* and *flhB* of *E. piscicida*. Relative gene expression (ΔΔCt) for *fimA* and *flhB* in *E. piscicida* in the presence of sub-IC of erythromycin. Values are the means ± standard deviations (error bars) from three independent experiments, with three technical replicates in each experiment. (**c**) cDNA agarose gel electrophoresis was performed after qRT-PCR. Picture is representative of three biological replicates. (**d**) Presence of type 1 fimbriae in *E. piscicida* was evaluated by immunoblotting with α-FimA in total cell lysate, secreted proteins, and shear cell fractions, both in the absence (−) and presence (+) of sub-IC of erythromycin. The GapA of *E. piscicida* was used as a reference protein. Picture is representative of three biological replicates.

Next, we performed RNA-seq to understand the genome-wide gene expression profile during the exponential phase when *E. piscicida* is exposed to the sub-IC of erythromycin. By applying the strict statistical cutoff (a false discovery rate [FDR] of 0.01 and a log2 fold change of >2 or <–2), a total of 397 genes (11% of whole genes encoded in *E. piscicida*) were found to be differentially expressed in the cells treated with the sub-IC of erythromycin compared to the non-treated control group. Particularly, 180 and 217 genes were up- and downregulated in the presence of the sub-IC of erythromycin, respectively (Fig. 2a; Table S1).

Furthermore, a functional enrichment analysis with the 397 differentially expressed genes (DEGs) was performed (Table S2). Whereas secondary metabolite biosynthesis, transport, and catabolism (Q) was the only functional category significantly enriched among upregulated genes, downregulated genes were significantly associated with three functional categories: energy production and conversion (C), amino acid transport and metabolism (E), and cell motility (N) (Table S2). These data suggest that erythromycin induces the bacteria to be sessile by reducing cell motility.

The expression levels of DEGs were further evaluated by qRT-PCR to validate the RNA-seq results. The expression level of *dnaJ* (ETAC_02795), which is a widely used gene in *E. tarda* as an internal control gene (44), was independent of the sub-IC of erythromycin and was used to normalize the gene expression level of target genes. Two genes (*fimA* and *flhB*) from the DEGs involved in the cell adhesion and cell motility that displayed the increased or decreased transcription levels in response to the sub-IC of erythromycin were chosen for validation (Table 1). *fimA* encodes an essential component protein for type 1 fimbria structure and cell shape. *flhB* is known as a critical gene that constitutes for the basal body of a flagellar apparatus used in cell motility. The expression level of *fimA* was increased about 3.3-fold, and the expression level of *flhB* was decreased by 1.8-fold when cells were exposed to the sub-IC of erythromycin (Fig. 2b and c). The expression levels of two representative genes were further investigated by qRT-PCR and gel electrophoresis imaging analyses, and the results were in good agreement with the RNA-seq data (Table 1; Fig. 2a through c). Taken together, the genome-wide transcriptomic analysis suggests that the sub-IC of erythromycin plays an important role in biofilm formation and cell motility.

**TABLE 1:** Expression levels of type 1 fimbria- and flagellum/chemotaxis-associated genes under sub-IC of erythromycin

Gene identifier	Annotation	Fold change (log2)
Type 1 fimbria-associated genes		
ETAC_RS04945	Type 1 fimbrial protein	4.45
ETAC_RS04950	Molecular chaperone	4.66
ETAC_RS04955	Fimbrial biogenesis outer membrane usher protein	4.14
ETAC_RS04960	Fimbrial protein	4.56
Flagellum/chemotaxis-associated genes		
ETAC_RS05840	Flagellar biosynthesis protein FlhB	−3.91
ETAC_RS05895	Flagellar basal body rod protein FlgG	−3.69
ETAC_RS05900	Flagellar basal body L-ring protein	−2.76
ETAC_RS05910	Flagellar rod assembly protein FlgJ	−3.71
ETAC_RS05915	Flagellar hook protein FlgK	−2.21
ETAC_RS06365	Flagellar motor protein MotA	−2.32
ETAC_RS06390	Methyl-accepting chemotaxis protein	−3.67
ETAC_RS06405	Chemotaxis protein CheY	−2.79
ETAC_RS06410	Chemotaxis protein CheZ	−2.97
ETAC_RS10075	Flagellar biosynthesis protein	−3.43
ETAC_RS10085	Flagellar hook-associated protein FliD	−3.07
ETAC_RS10160	Flagellar motor switch protein FliM	−2.99
ETAC_RS10125	Flagellar MS-ring protein	−5.41

### Increased biofilm formation by the sub-IC of erythromycin

From the RNA-seq analysis, the genes (ETAC_04905, ETAC_04910, and ETAC_04915) involved in the type 1 fimbria biosynthesis were identified to be upregulated (from 17- to 26-fold, Table 1) when cells were exposed to the sub-IC of erythromycin. To validate the transcriptomics results, we identified the protein level of FimA, encoded by ETAC_04905, by an immunoblotting experiment with and without the sub-IC of erythromycin treatment. The protein band detected by anti-GapA which was used as an internal control protein was observed regardless of erythromycin. The FimA protein band was only detected in the sub-IC of erythromycin supplemented medium (Fig. 2d). The results from both transcriptional and translational approaches support that FimA is produced from the high expression and the synthesis of type 1 fimbriae. The synthesis of type 1 fimbriae could be promoted when *E. piscicida* is exposed to the sub-IC of erythromycin.

**Fig 3:**
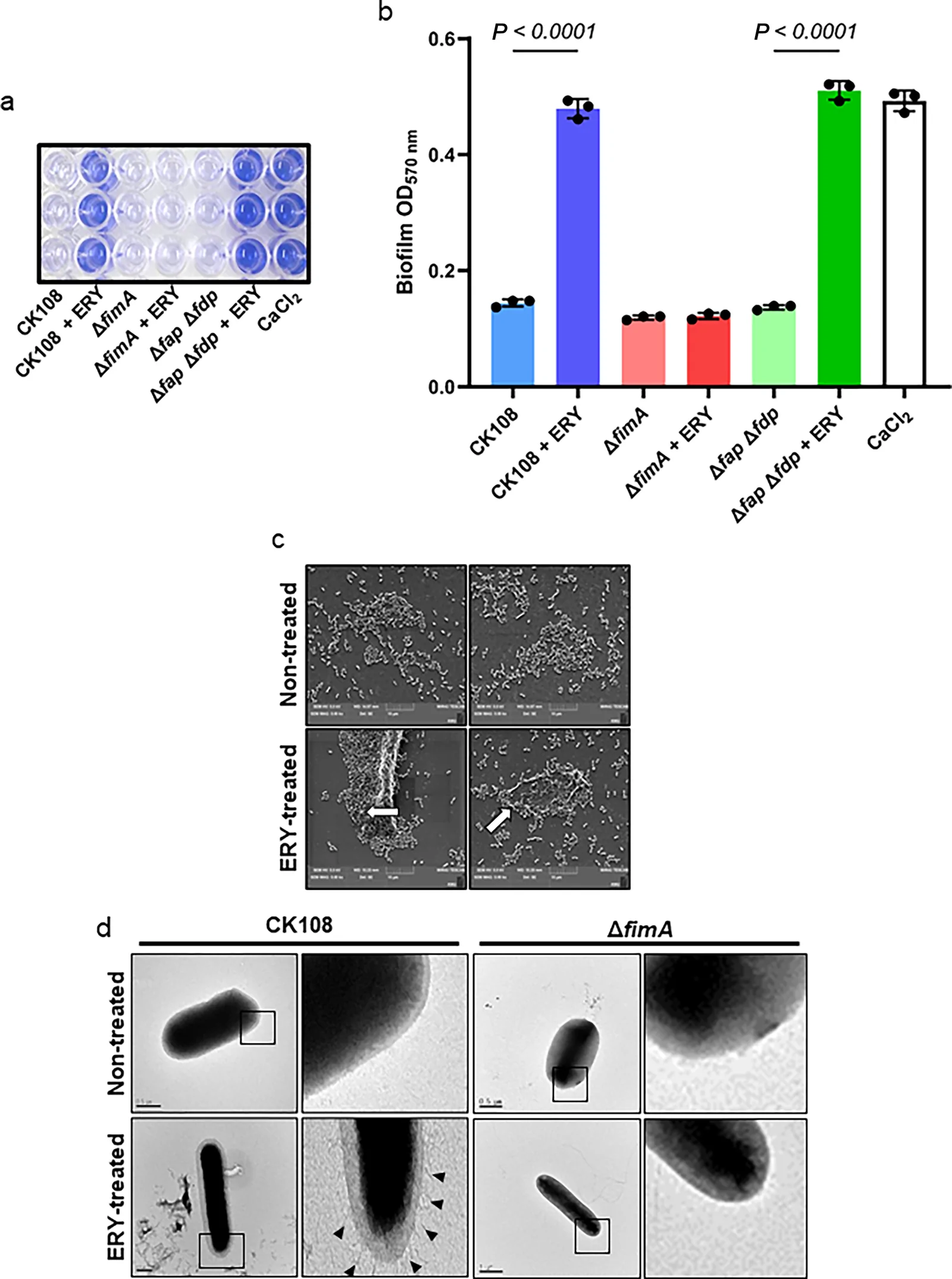
Confirmation of the increase in fimbria-mediated biofilms of *E. piscicida* with sub-IC of erythromycin. Sub-IC of erythromycin increases biofilms formed by *E. piscicida*. (**a**) Representative crystal violet assay image of *E. piscicida* strains on polyethylene surfaces. The bioﬁlms were stained with crystal violet and dissolved in 95% ethanol solution. The *E. piscicida* Δ*fimA* mutant, and Δ*fap, fdp* mutant were used as controls. A positive control for biofilm formation was established by supplementing the medium with 20 mM calcium chloride. (**b**) Result of crystal violet elution with 95% ethanol. Biofilm formation was quantified by measuring the OD_570 nm_. Values are the means ± standard deviations (error bars) from three independent experiments, with three technical replicates in each experiment. The *P* values indicate on top, according to independent sample *t*-tests (one-tailed hypothesis). (**c**) Field emission-scanning electron microscope (FE-SEM) images of *E. piscicida*, grown in absence (Non-treated) or in presence (ERY-treated) of sub-IC erythromycin. The white arrows indicate the biofilm matrix attached to the bacteria. (**d**) Transmission electron microscope (TEM) images of *E. piscicida*, grown in absence (Non-treated) or in presence (ERY-treated) of sub-IC erythromycin. The black boxes indicate the enlarged sections, which is shown in the right panel. The black arrowheads indicate the intact type 1 fimbriae on *E. piscicida* surface.

A crystal violet assay was adopted to show whether *E. piscicida* produces biofilm by the fimbria-mediated mechanism when erythromycin is present at sub-IC. To test this hypothesis, we constructed strains of *fimA* (major type 1 fimbrial protein) deletion. To make sure that this reduced biofilm formation is not affected by flagellum-mediated motility, we also used the CK248 strain, which is the *fap/fdp* (*fap*, flagellin-associated protein; *fdp*, flagellin domain-containing protein) double knockout mutant as a control (45). As shown in Fig. 3a and b, compared to the culture of *E. piscicida* without CaCl_2_, strong purple color was observed from the culture with CaCl_2_ which is a biofilm generator chemical agent (22, 46). A robust crystal violet signal similar to the one in *E. piscicida* culture with CaCl_2_ was detected in culture treated with the sub-IC of erythromycin in both CK108 and CK248 strains (Fig. 3a and b), and an almost clear crystal violet signal was observed from the CK248 strain with the sub-IC of erythromycin. Inhibition of flagellum-mediated motility is generally associated with a transition from a motile to a sessile lifestyle, which can facilitate biofilm formation in many bacteria (47). However, the results of this study indicate that both *fap* and *fdp* flagellin genes analyzed here are not directly involved in biofilm formation in *E. piscicida*. Interestingly, the purple color was hardly observed from the *fimA* deletion strain regardless of the addition of erythromycin (Fig. 3a and b). This result confirms that *fimA* plays a critical role in the biofilm formation (48–50).

Field emission scanning electron microscopy (FE-SEM) was used to observe the microbial community and cell morphology changes when cells were subjected to the presence and absence of the sub-IC of erythromycin. There was no significant cellular community formation in the absence of erythromycin, but bacterial clumps were observed when cells were treated with the sub-IC of erythromycin (Fig. 3c). Exopolysaccharides are also one of the agents that facilitate cell aggregation and biofilm formation (20, 51). To test if the observed biofilm is mediated by exopolysaccharides production when the sub-IC of erythromycin is present, a Congo red agar plate assay was performed (Fig. S1). Congo red-supplemented BHI medium primarily detects the production of Congo red-binding extracellular polysaccharides, which represent one component of the extracellular polymeric substances (EPS) matrix. Therefore, this assay evaluates polysaccharide-associated biofilm characteristics rather than fimbria-mediated biofilm formation nor overall EPS production. In contrast to the black color formation in the control strain of *Staphylococcus epidermidis* without erythromycin treatment, no color change was observed in *E. piscicida* regardless of erythromycin treatment. This suggests that the biofilm production by *E. piscicida* strain is exopolysaccharides-independent. A transmission electron microscopy (TEM) was used to observe individual cell morphologies. While there was no noticeable apparatus in the absence of erythromycin, numerous filamentous structures which may be type 1 fimbriae were identified in the non-erythromycin treated cells (Fig. 3d). These results suggest that at its sub-IC, erythromycin stimulates the expression and protein levels of FimA, cell aggregation, and biofilm formation in *E. piscicida*.

### Reduced motility by the sub-IC of erythromycin

**Fig 4:**
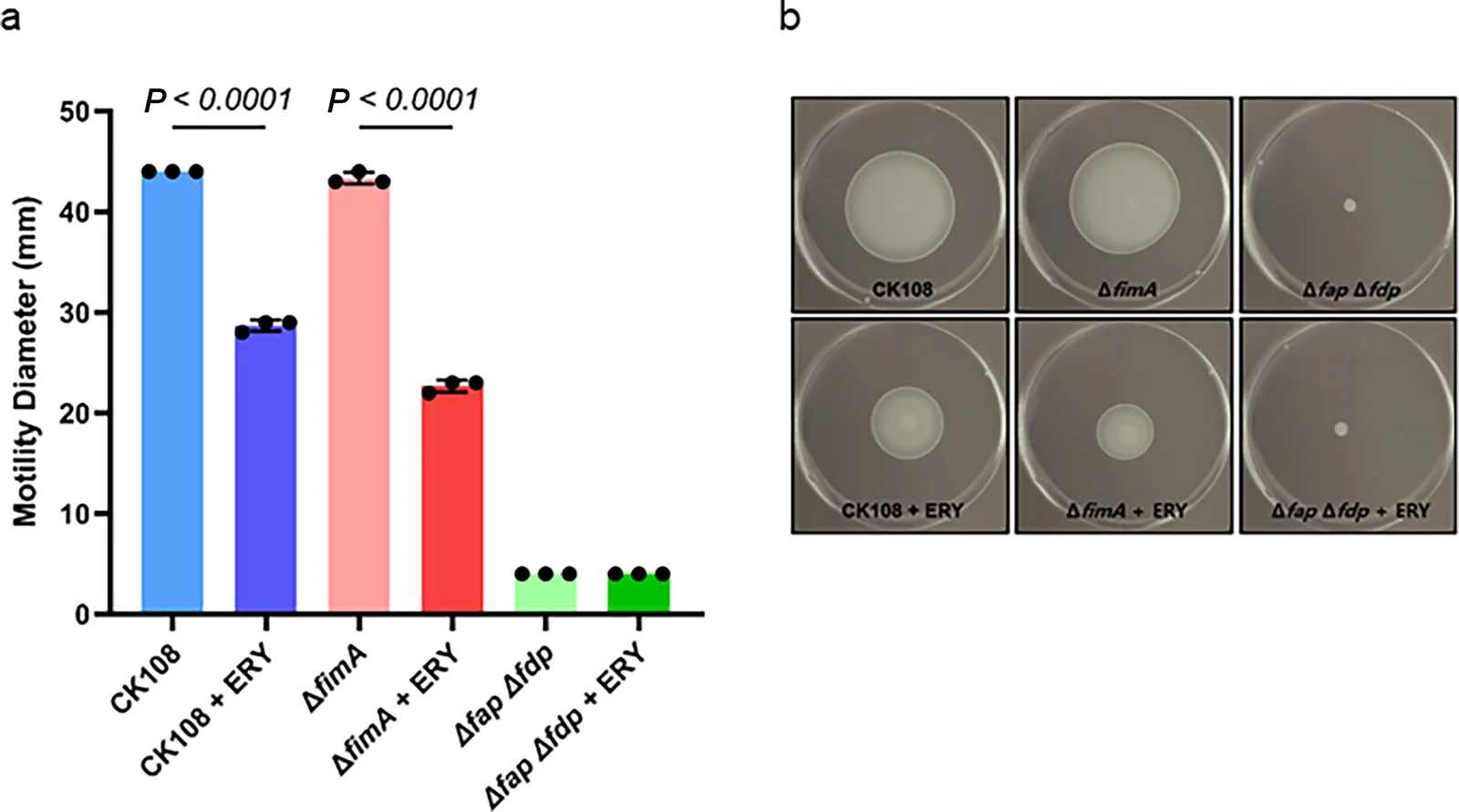
Effect of sub-IC of erythromycin on motility of *E. piscicida. E. piscicida* was cultured in BHI medium to an OD_600 nm_ of 0.3, and 3 µL culture was spotted onto the soft agar with or without sub-IC of erythromycin. The plates were incubated for 20 h at 27°C. (**a**) Graph indicating the diameter of swimming zone. Values are the means ± standard deviations (error bars) from three independent experiments, with three technical replicates in each experiment. The *P* values indicate on top, according to independent sample *t*-tests (one-tailed hypothesis). (**b**) Representative soft-agar motility assay image of *E. piscicida* strains. The *E. piscicida* Δ*fimA* mutant, and Δ*fap, fdp* mutant were used as controls.

Many bacteria are motile through flagella and *E. piscicida* is also predicted to possess the flagellum encoding genes (45). Interestingly, in contrast to the type 1 fimbrial biosynthesis genes, all the identified DEGs involved in cell motility (*flgB*, *flgC*, *flgJ*, *flgG*, *flgK*, *flhA*, *flhB*, *fliD*, *fliG*, *fliM*, *fliN*, *fliS*, *fliT*, and *motA*) were downregulated under the sub-IC of erythromycin (Table 1). A motility assay was performed to validate the transcriptome results. To synchronize the growth phase and initial cell density, *E. piscicida* strains were cultured to an OD_600 nm_ of 0.3, and the cultures were subsequently drop-spotted onto soft agar plates containing erythromycin at sub-IC. The cellular swimming distance was measured with wild-type, *fimA* deletion, and *fap/fdp* double knockout strains. While the *fap/fdp* double knockout strain was not motile, the movement of wild-type was decreased (about 35%) by the sub-IC of erythromycin (Fig. 4). This proves that the genome-wide gene expression data are compatible with the phenotype and erythromycin inhibits the motility of *E. piscicida*. We also tested the *fimA* deletion strain by the motility assay and found that the reduced motility is caused by the sub-IC of erythromycin, suggesting that the cellular motility is *fimA*-independent.

### Increased rate of cell adhesion and invasion by the sub-IC of erythromycin

**Fig 5:**
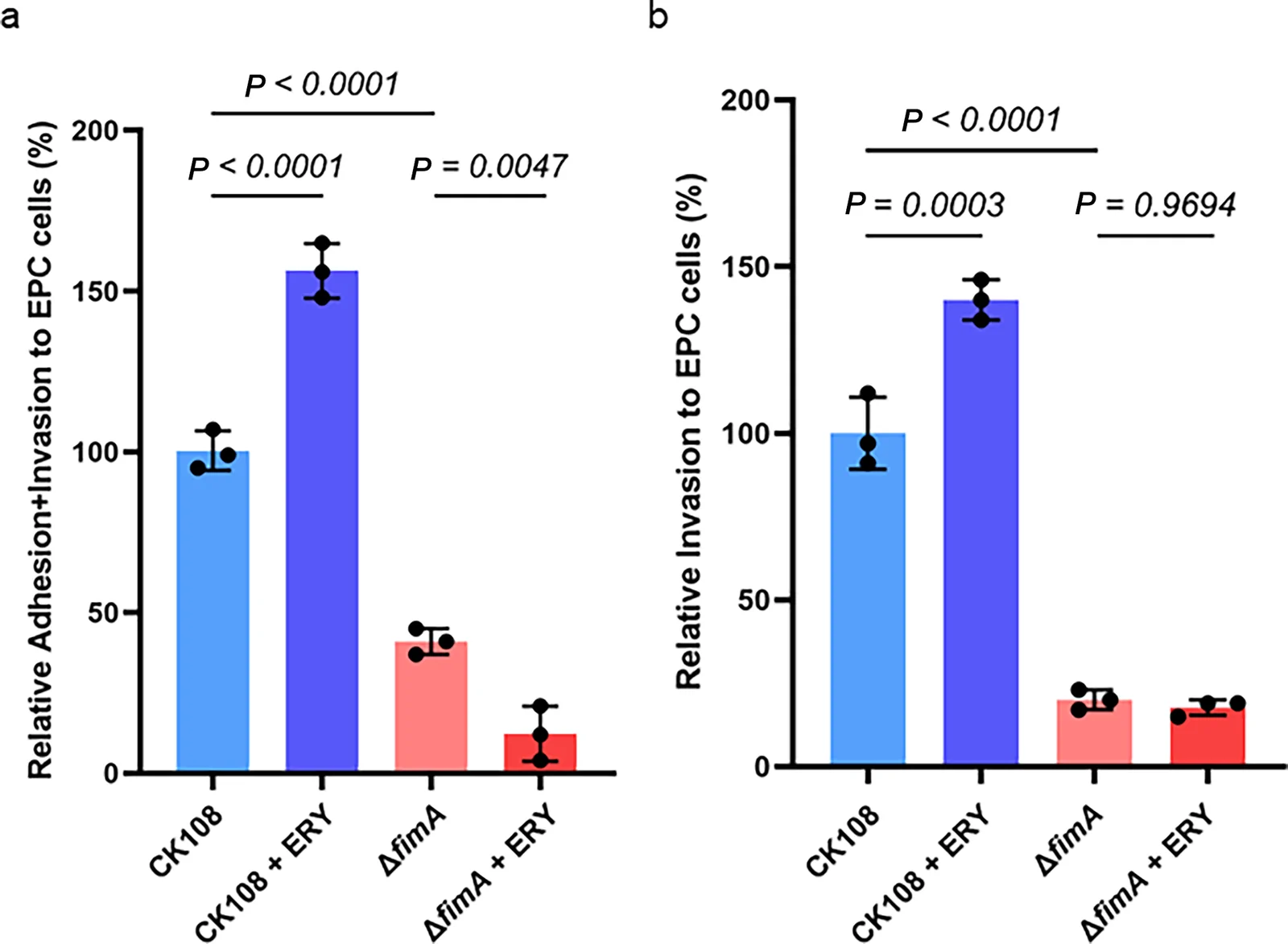
Adhesion and invasion assays of *E. piscicida* with or without sub-IC of erythromycin. Adhesion and invasion assays were compared by setting the relative means to 100% of WT. (**a**) Relative percentage of EPC cell-associated bacteria. (**b**) Relative percentage of invaded bacteria to EPC cells. Values are the means ± standard deviations (error bars) from three independent experiments, with three technical replicates in each experiment. The *P* values indicate on top, according to independent sample *t*-tests (one-tailed hypothesis).

Since *E. piscicida* is a notorious fish-pathogen isolated from diseased flounder fish (52), we aimed to further explore the pathogenicity of *E. piscicida* under the sub-IC of erythromycin. Based on the results from the transcriptome analysis and the phenotypic effect of erythromycin on cell motility and biofilm formation, we hypothesized that *E. piscicida* could be more pathogenic to fish in the sub-IC of erythromycin than in an antibiotics free aquatic environment. *E. piscicida* was grown with and without the treatment of sub-IC of erythromycin and then infected to EPC cells followed by measurement of the capability of adhesion and invasion. Compared to the non-treated condition, 50% more wild-type cells invaded and internalized the EPC cells in the presence of sub-IC of erythromycin (Fig. 5). In the wild-type cells, the *fimA* deletion strain count was found to reduce to half. Surprisingly, the number of *fimA* deletion strain inside EPC cells decreased to about fourfold after the supplementation of the sub-IC of erythromycin during the *E. piscicida* growth (Fig. 5). Overall, these data suggest that the fish pathogen prefers to invade and colonize inside the host cells rather than wander when erythromycin is present, by promoting the synthesis of type 1 fimbriae.

### Increased fatality by the sub-IC of erythromycin using *in vivo* zebrafish infection assay

**Fig 6:**
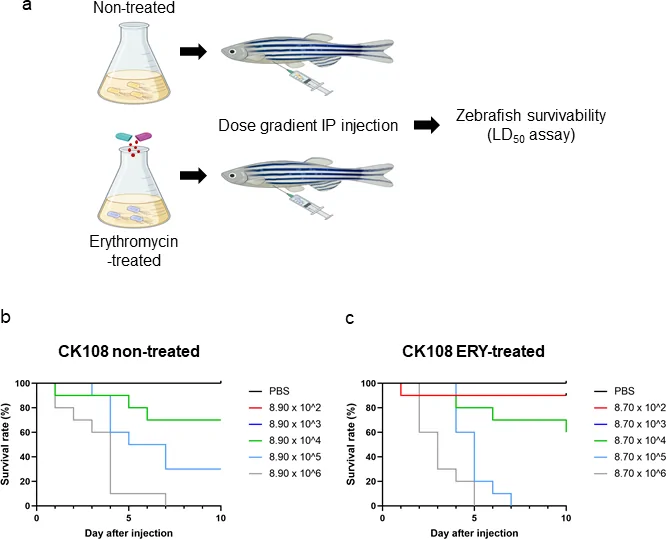
Zebrafish *in vivo* virulence assay about sub-IC of erythromycin on *E. piscicida. E. piscicida* strains were cultured with or without 5 µg/mL erythromycin to OD_600 nm_ of 0.8. (**a**) A brief scheme for the processes of the zebrafish *in vivo* virulence assay. (**b**) Survivability of zebrafish injected with 20 µL of CK108 at doses of 8.90 × 10^2-6^ CFU that cultured without erythromycin. (**c**) Survivability of zebrafish injected with 20 µL of CK108 at doses of 8.70 × 10^2-6^ CFU that cultured with 5 µg/mL of erythromycin. All zebrafish and mortality rates were monitored for at least 10 days daily, after injection.

Biofilm is known as a key virulence factor in pathogenic bacteria (51). While the biofilm formation and cell invasion rates were increased by the sub-IC of erythromycin (Fig. 5), it has not yet been determined how the increased biofilm formation causes damage to the host. To determine the *in-vivo* pathogenicity and LD_50_ value, an infection assay using zebrafish was used. In the assay, a series of 10-fold increasing doses of non-treated and the sub-IC of erythromycin treated wild-type *E. piscicida* were administered. The survival in three study groups of zebrafish (10 of the non-treated group, 10 of the group with treatment of sub-IC of erythromycin, and 10 of control with PBS buffer treatment) was monitored for 10 days after the intraperitoneal injection (Fig. 6a). A dose-dependent mortality was exhibited with significant death of zebrafish at *E. piscicida* threshold of 8.70–8.90 × 10^6^ CFU (Fig. 6b and c). The LD_50_ for the *E. piscicida* treated with the sub-IC of erythromycin (1.28 × 10^5^ CFU) was 2.2-fold higher than that of the non-treated *E. piscicida* (2.81 × 10^5^ CFU) (Table 2). An *in-vivo* survival assay revealed that the sub-IC of erythromycin increases the virulence of *E. piscicida* in zebrafish.

**TABLE 2:** LD_50_ *E. piscicida* strain in zebrafish

Strain	LD_50_ values (CFU/fish)	*P* value
*E. piscicida* CK108	2.81 × 10^5^ CFU	*P* ≤ 0.0001
*E. piscicida* CK108 treated erythromycin 5 µg/mL	1.28 × 10^5^ CFU	

## DISCUSSION

Global fish consumption has shown a steady increase over the past decades. According to FAO statistics, per capita fish intake rose from approximately 9 kg in 1961 to 20.6 kg in 2021, reflecting an average annual growth rate of about 3.1%. Based on these current trends, per capita consumption is projected to reach nearly 21.3 kg by 2032 (53). In parallel, the global demand for meat has continued to rise, intensifying concerns regarding the depletion of food resources. Within this context, fish have emerged as a critical source of animal-derived protein. Projections indicate that global demand for fish, which was estimated at 59 million tonnes in 2020, is expected to increase by nearly 74% and reach approximately 103 million tonnes by 2050. Importantly, more than three billion people are expected to depend on fish as a primary source of animal protein (54). Ensuring a sustainable and healthy food supply is, therefore, recognized as a global priority, and this recognition has heightened the importance of aquaculture (farm-raised fisheries) over traditional capture fisheries (wild-caught), as highlighted by FAO (55) and recent analyses (55, 56).

In aquaculture systems, outbreaks of infectious diseases are shaped by the interplay of environmental factors, host susceptibility, and pathogenic microorganisms. Among the wide range of pathogens—including bacteria, viruses, fungi, and parasites—bacterial infections account for more than 53% of documented cases (57). *Edwardsiella piscicida*, the pathogen investigated in this study, is a notorious fish pathogen that causes severe diseases such as septicemia and edwardsiellosis in economically important cultured species such as flounder and catfish (7, 44, 45, 58, 59). To combat this bacterial infection, various types of antibiotics treatment methods are used in the current aquaculture industry. One of the treatments includes an oral administration of a mixture of feedstock and antibiotics to the fish. Since the accumulation of residual antibiotics in fish after such treatment may have a negative effect on humans who consume such treated fish, the method of mixing the antibiotics with feed is prohibited worldwide (60). Another method of introduction of antibiotics in the aquatic environment is through their delivery in diluted concentrations directly in aquatic cultures. This method can threaten the aquatic environment because the attenuated concentrations of antibiotics in aquaculture can reach to the sub-inhibitory concentrations (sub-IC) and cause unknown effects. The sub-IC of antibiotics does not affect the growth of bacteria; however, it causes abnormal gene expression in pathogenic bacteria (52). The diluted concentration of antibiotic cannot inhibit the propagation of pathogens but can act as a signal molecule that unexpectedly alters gene expression. For example, the sub-IC of amoxicillin, lincomycin, and oxytetracycline is reported to increase the biofilm of S*treptococcus suis* (32). The sub-IC of penicillin is reported to increase the biofilm and hydrophobicity of *Corynebacterium diphtheriae* (33)*.*

Biofilm formation is a critical determinant of bacterial adhesion to host tissues and a key virulence attribute in the early stages of infection (51). Capability of cell adhesion to the host surface is very important in the early stages, as a virulence factor (61). Pathogenic bacteria initially adhere to the surface of host by increasing their population and producing biofilms and causing fatal effects to the host (20, 51). Pathogenic bacteria cause enormous economic losses by infecting host fish. The pathogenesis of *E. piscicida* appears to be multifactorial virulence factors such as a type III secretion system (T3SS), type VI secretion system (T6SS), flagellin, and two-component systems (52). Many research studies have also shown that multiple gene regulated biofilm formation is another critical factor that enables *E. piscicida* pathogenicity (62–72). EseC, a component of T3SS, affects the synthesis of biofilm by EseE acting as a regulator and is also regulated by sigma factors RpoS and RpoN. In addition, LuxS involved in quorum sensing controls the expression of genes involved in the biofilm formation. HutZ and TrxH also play a role in the production of biofilm (62, 66, 72). In addition, bacterial biofilm formation that induces cellular adhesion is responsive to other environmental factors such as pH, nutrient, and osmotic pressure (21, 22). These studies mainly conducted *in vitro* assays to identify the role of biofilm in pathogenesis using the biofilm-defect mutants (73). There are limited studies that report the investigation of regulation of pathogenicity through biofilm formation in wild-type strains. To address this gap, the regulation of pathogenicity by biofilm formation was studied through a comprehensive approach including *in silico*, *in vitro*, and *in vivo* approaches in this study.

This study addressed the hypothesis that sub-IC of aquaculture-associated antibiotics, which rapidly disperse and dilute in aquatic environments, may act as signaling cues that alter the expression of key virulence determinants in pathogenic bacteria and thereby modulate disease susceptibility of fish hosts. In line with this idea, two potential scenarios were considered. In one, exposure to sub-inhibitory antibiotic levels increases virulence factor expression, producing hypervirulent strains capable of inflicting greater damage on the fish host even at similar bacterial loads. In the other, virulence factor expression decreases, resulting in a transiently attenuated phenotype that evades host immunity through a commensal-like camouflage and persists in higher numbers; upon withdrawal of the antibiotic cue, virulence traits could be restored, causing sudden host damage. Both scenarios imply that environmental antibiotic signals may reshape pathogen virulence with serious implications for aquaculture losses.

To test this, we first established sub-IC of erythromycin for *E. piscicida* and analyzed transcriptomic changes using RNA-seq. Notably, type 1 fimbriae—key virulence determinants involved in adhesion and biofilm formation—were significantly upregulated. Previously, only salt concentration had been identified as an environmental regulator of fimbrial expression in *Edwardsiella* (74). That study showed increased fimbrial production and hemagglutination in an *E. tarda* strain from Japanese flounder under high-salt (3% NaCl) versus low-salt (0% NaCl) conditions. However, because that strain naturally infects marine species, high-salt exposure corresponds to its usual habitat and may not adequately represent a hypervirulence trigger. Our findings demonstrate that, in addition to salinity, sub-IC of erythromycin can act as an environmental cue modulating fimbrial expression in *Edwardsiella*. Similar to reports with other pathogens, such fimbrial upregulation suggests enhanced adhesion and pathogenic potential through increased biofilm formation. Concomitantly, we observed the downregulation of flagellar and chemotaxis-related genes, consistent with the known shift from motility to surface attachment during biofilm development. Together, these transcriptomic changes point to a phenotypic transition toward stronger biofilm formation under low-dose erythromycin.

Building on the *in silico* results, we verified these predictions experimentally. Crystal violet staining and electron microscopic analyses confirmed that type 1 fimbriae directly mediate biofilm formation and that exposure to sub-IC of erythromycin markedly increased biofilm biomass. The type 1 fimbrial *fimA* homologs are widely present in various Gram-negative pathogens, similar biofilm modulation by sub-IC of erythromycin may occur in other *fimA*-positive bacteria, while the mechanisms vary depending on the species or conditions. Thus, although our results are limited to *E. piscicida*, we suggest sub-IC of erythromycin may promote *fimA*-associated biofilm in other *fimA*-carrying pathogens.

Additionally, reduced motility due to decreased expression of flagellar and chemotaxis-related genes was corroborated by soft-agar assays, validating the transcriptomic predictions. Infection assays using cell-lines revealed that *E. piscicida* exposed to sub-IC of erythromycin displayed enhanced adhesion and invasion compared with untreated controls. Consistent with these findings, zebrafish (*Danio rerio*) infection experiments showed a lower LD₅₀ for sub-IC of erythromycin exposed *E. piscicida*, suggesting that increased fimbrial expression translated into heightened virulence and greater host susceptibility *in vivo*. Specifically, infection with erythromycin pre-exposed bacteria resulted in 100% mortality by day 7, whereas approximately 30% of hosts survived until day 10, the end of the study, when infected with non-exposed wild-type bacteria. This pronounced difference in survival at comparable doses is biologically significant and may have important implications for disease severity and potential economic impact in aquaculture settings.

**Fig 7:**
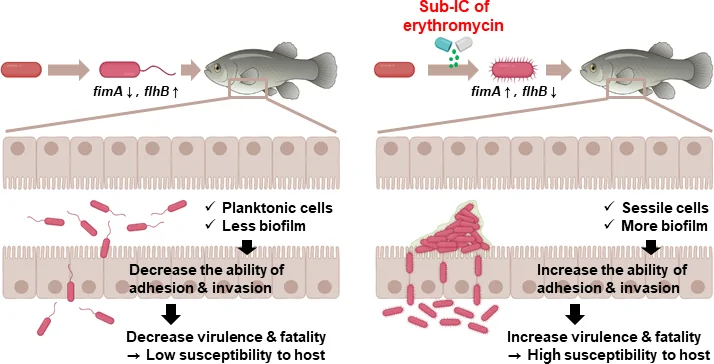
Schematic diagram illustrating the proposed model of *E. piscicida* pathogenesis of fish host infection as derived from sub-IC of erythromycin exposure. In comparison to the normal conditions without antibiotic (erythromycin in this case) pollution, *E. piscicida* adapted to sub-IC of antibiotic discharged into the environment exhibits an increased the fimbria-mediated biofilm formation and reduced motility. This erythromycin-adapted fish pathogen displays in a sessile state within fish hosts, and their adhesion and invasion abilities to host intestinal epithelial cells are enhanced. This leads to an increase in colonization capability of pathogen, and with the upregulation of biofilm formation by exposure to sub-IC of erythromycin, the host susceptibility is increased. Thus, even when the fish hosts exposed to a low quantity of pathogenic bacteria, it can be showing a high fatality rate.

Collectively, our study shows that *E. piscicida*, a major bacterial pathogen in aquaculture, perceives low-level antibiotic stress as an environmental signal that reconfigures a major biofilm phenotype and its associated virulence machinery. Specifically, sub-IC of erythromycin increased expression of the key adhesin type 1 fimbriae while suppressing flagellum-mediated motility and chemotaxis-related genes, promoting adaptation to a sessile, biofilm-forming lifestyle better suited for host colonization. This dual shift enhanced epithelial adhesion and invasion and ultimately increased host mortality, experimentally demonstrating that environmental cues can recalibrate virulence factor expression and modulate host susceptibility even in wild-type strains. The work, thus, provides evidence that environmental antibiotics—at concentrations far below therapeutic levels used in aquaculture systems—may alter the pathogenic potential of common aquaculture bacteria, with direct implications for fish health and mass mortality events (Fig. 7).

Importantly, unlike many previous studies that relied on biofilm-defective mutants, our experiments employed wild-type *E. piscicida* to capture naturally occurring responses to environmental conditions. By documenting antibiotic-driven increases in biofilm and fimbrial expression and linking them to enhanced host-cell attachment, invasion, and disease outcomes, we establish a mechanistic basis for how low-level antibiotic residues can shift pathogen behavior. This experimentally validates the long-suspected risk of indiscriminate antibiotic use in aquaculture and provides a scientific foundation for developing evidence-based guidelines and regulatory standards for antibiotic application in fish farming.

## MATERIALS AND METHODS

### Bacterial strains, plasmids, and culture conditions

Strains and plasmids used in this study are listed in Tables S3 to S5. *Edwardsiella piscicida* CK108 was grown at 27°C in brain heart infusion (BHI) medium (Difco, USA). *Escherichia coli* strains were cultured in Luria broth (LB) medium (Difco, USA) at 37°C. When required, the medium was supplemented with appropriate antibiotics at the following concentrations: ampicillin 100 µg/mL, chloramphenicol 30 µg/mL, diaminopimelic acid 50 µg/mL, and erythromycin at 5 µg/mL, respectively.

### Bacterial growth monitoring

Kinetics of cell growth were measured by using an Epoch2 microplate spectrophotometer (BioTek, USA) at 600 nm wavelength. Briefly, erythromycin was diluted sequentially in BHI medium and placed into a 96-well cell culture plate (SPL, Republic of Korea). The 1% of overnight cultured *E. piscicida* was inoculated into 200 µL of medium. Cell growth was measured every 30 min for 16 h at 27°C under a double orbital-shaking condition in a microplate reader. For comparison of specific growth rates, *E. piscicida* was cultured with erythromycin (from 5 to 25 µg/mL) until exponential (7 h) and stationary phase (17 h), respectively. Each culture was 10-fold diluted and spotted onto fresh BHI medium for CFU measurements.

### Biofilm assay

Biofilm quantifications were performed after 24 h of growth in BHI medium with or without 5 µg/mL of erythromycin. About 1% cultures of *E. piscicida* were inoculated into BHI media for overnight duration. Cultures were incubated in polyethylene tubes (SPL, Republic of Korea) for 24 h at 27°C with 200 rpm shaking. Cultures in the tube were removed and rinsed with distilled water, followed by staining with 1% (wt/vol) crystal violet (BD BBL, USA). The tubes were rinsed with distilled water, and 95% of ethanol was used to dissolve the stained biofilm. Biofilm formation was quantified by measuring the dissolved solution (200 µL) at 570 nm absorbance by using an Epoch2 microplate Spectrophotometer.

### Motility assay

Strains of *E. piscicida* were cultured in BHI medium to an OD_600 nm_ of 0.3, and 5 µL of cell suspensions was spotted onto the middle area of soft agar plates (0.3% [wt/vol]) with and without erythromycin (5 µg/mL). After 20 h of incubation at 27°C, the motility was assessed by examining the diameter of the movement.

### Congo red agar assay

The solid plate (1% [wt/vol] agar) of BHI medium containing 0.08% Congo red was supplemented with or without 5 µg/mL of erythromycin. Overnight cultures of *E. piscicida* and *Staphylococcus epidermidis*, as a positive control, were streaked onto plates and incubated at 27°C, followed by observation at 24 and 48 h after the cell inoculation.

### TCA precipitation and SDS-PAGE for proteins

To precipitate secreted proteins from *E. piscicida*, the cell culture was spun down, and 25% of trichloroacetic acid (TCA), at a final concentration of 7%, was added to the supernatant. The precipitated protein was pelleted by centrifugation at 10,000 × *g* for 10 min at 4°C and washed twice with acetone to remove the remaining TCA. After drying the pellet completely, it was dissolved in 2× protein digestion buffer and then separated by sodium dodecyl sulfate-polyacrylamide gel electrophoresis (SDS-PAGE). Coomassie Brilliant Blue G-250 (Sigma, USA) was used for protein staining.

### Immunoblotting analysis

After the protein samples were separated by SDS-PAGE, proteins in polyacrylamide gels were transferred to a nitrocellulose membrane (Thermo Fisher Scientific, USA) by a semi-dry transfer device (Mini Trans-blot Electrophoretic Transfer cell, Bio-Rad, USA) using Towbin’s buffer (TB) (25 mM Tris, 192 mM glycine, 20% [vol/vol] methanol, pH 8.3). The membrane was blocked in Towbin’s saline buffer (TBS) containing 5% skim milk for 2 h and incubated with the diluted (1:100) FimA polyclonal primary antibody at 4°C for 8 h. The membrane was washed several times with TBS. The secondary antibody of horseradish peroxidase-conjugated goat anti-rabbit IgG (Invitrogen, USA) was diluted (1:5,000) with TBS containing 5% skim milk and incubated with the membrane for 2 h. Protein bands on the membrane were detected by the fluorescence imaging system (Korea Lab Tek, Republic of Korea) after several times of washing with TBS. The anti-FimA polyclonal antibody was generated in this study (Fig. S2), and the anti-GapA polyclonal antibody was kindly provided by Dr. Kang at Pusan National University (75).

### Electron microscopy analyses

In order to observe the biofilm formation of *E. piscicida* via scanning electron microscopy (SEM), 9 mm coverslips were incubated with *E. piscicida* exposed to sub-IC antibiotics treatment. Formed biofilm on coverslips was fixed with 2.5% glutaraldehyde (Sigma, USA) resolved in PBS (pH 7.4) for 4 h at room temperature. The fixed samples were dehydrated with graded acetone solutions of 10%, 30%, 50%, 70%, 90%, and 100% for 15 min, subsequently. Completely dried samples were coated with 5 nm thick platinum using Q150R S (Quorum Technologies, UK). MIRA-3 SEM (Tescan, Czechia) was used to observe the biofilm morphology at accelerating voltage of 5 kV.

To conduct transmission electron microscopy (TEM) analysis, *E. piscicida* was incubated at 27°C for 24 h in BHI medium with or without 5 µg/mL of erythromycin. Bacterial cells were harvested and diluted using TBS buffer. The bacterial cells were placed on carbon coated copper 200 mesh grids (Electron Microscopy Sciences, USA), and the grids were placed on 50 µL of the cell suspension for 30 s to fix the cells. Grids were washed three times with distilled water. Completely removing the water, proceed with negative staining using 1% uranyl acetate. After staining and washing with distilled water three times, the samples were dried for 2 h at room temperature. Prepared grids were visualized by H-7500 (Hitachi Ltd., Japan) operated at 120 kV.

### Quantitative reverse transcriptase-polymerase chain reaction (qRT-PCR)

qRT-PCR was performed to determine the relative expression level of genes under the Sub-IC of erythromycin. *E. piscicida* CK108 was grown in BHI broth at 27°C with 200 rpm for 16 h, and total RNA was extracted from the culture by an AccuPrep Universal RNA Extraction Kit (Bioneer, Republic of Korea) according to the manufacturer’s instructions. The extracted RNA was treated by DNase I (Invitrogen, USA) to prevent DNA contamination. OneStep qRT-PCR Master Mix (Biofact, Republic of Korea) was used under the following amplification conditions: initial steps for 30 min at 50°C and for 5 min at 95°C, followed by 40 cycles of 20 s at 95°C, 30 s at 50°C, 20 s at 72°C, and finally 15 s at 95°C, 1 min 60°C, and 15 s at 95°C. The primer sets of *fimA*, *flhB*, *dnaJ* (endogenous control) for qRT-PCR were shown in Table S5. The expression of relatively different RNA was calculated by ΔΔCt method based on the expression level of a house keeping gene *dnaJ*. Sequentially, the products of qRT-PCR were electrophoresed to represent gene expression levels as differences in band thickness in agarose gel.

### Experimental animals

Adult zebrafish (*Danio rerio*) were procured from commercial aquarium, Gwang-won aquarium (Busan, Republic of Korea). The average weight (249.4 ± 55 mg) and average length (2.96 ± 0.2 cm) of the zebrafish used in this study were 0.29 ± 0.05 g and 3.06 ± 0.13 cm, respectively. The fish were acclimatized for 2 weeks in the aquatic housing system (21 Century High Tech, Daejeon, Republic of Korea), with the tank water filtered by reverse osmosis and photoperiod of 14 h light/10 h dark. The housing water parameters were monitored daily to maintain the following conditions: water temperature 27 ± 1℃, pH 7.5 ± 0.3, and conductivity 700 ± 50 μS/cm. During the acclimatization, fish were fed with a commercial diet (PRODAC, Cittadella, PD, Italy), twice per day. The fish were fasted for at least 12 h prior to each experiment.

### Infection of zebrafish

Zebrafish, *Danio rerio* (Kwang Won aquarium, Busan, Republic of Korea), were raised in an aquarium system constantly maintaining fresh water conditions. Zebrafish were anesthetized with tricaine methane sulfonate (Sigma, USA) prior to intraperitoneal (IP) injection. *E. piscicida* CK41 strain was cultured in BHI with or without 5 µg/mL erythromycin until the OD_600 nm_ reached a value of 0.8. Harvested bacteria were washed three times with phosphate buffered saline (PBS) and prepared to reach concentrations 10^3–6^ CFU/mL in PBS buffer. Resuspended cells in PBS were counted by culture on BHI agar plates after serial dilution to confirm the cell number. IP injection was performed in the abdominal cavity of zebrafish. The number of bacteria injected into each group of 10 zebrafish is as follows. Group 1 was injected with 20 μL of 8.90 × 10^3-6^ CFUs/mL *E. piscicida* CK108 wild-type. Group 2 was injected with 20 μL of 8.70 × 10^3-6^ CFUs/mL *E. piscicida* CK108 exposed to 5 μg/mL of erythromycin. Group 3 was injected with 20 μL of PBS used as a control. All zebrafish were monitored for at least 10 days post‐injection, and their mortalities were calculated to determine the LD_50_ value.

### RNA-seq preparation and data analysis

A preparation method for RNA-seq was followed as per our previous published study (52). Briefly, RNA was extracted by AccuPrep Universal RNA Extraction Kit (Bioneer, Republic of Korea) according to the manufacturer’s manual with additional DNA removal by Turbo DNA-free kit (Invitrogen, USA) when cells reached the exponential phase (OD_600 nm_ 0.6–0.8) in the presence or absence of 5 µg/mL erythromycin. The purity and integrity of RNA samples were assessed by measuring the absorbance at 260/280 nm using an Epoch2 microplate Spectrophotometer and Bioanalyzer 2100 (Agilent Technologies, USA). Ribosomal RNA from the purified RNA (RIN > 8) was removed by the AnyDeplete probe, and the remaining RNA was used to construct sequencing library by Truseq Stranded Total RNA H/M/R Prep Kit (Illumina, USA) according to the manufacturer’s recommendations. The quality of the amplified libraries was verified by Bioanalyzer (Agilent, USA). NovaSeq 6000 (Illumina, USA) platform was used for sequencing and 100-bp of pair-end reads were generated (20–30 million reads per sample).

To analyze raw RNA-seq data, raw reads were initially assessed for their sequencing quality by FastQC (76). Poor quality or short reads (<10 bp) were trimmed by Cutdadpt (77). The preprocessed reads were aligned to the reference genome of *E. piscicida* C07-087 by Bowtie2 (78). Mapped reads to each transcript were counted by HTSeq (79), and DESeq2 (80) was used to identify differentially expressed genes (DEGs). Genes with log2 fold-change larger than 2 and false discovery rate (FDR) by Benjamini-Hochberg (BH) correction for multiple hypothesis testing less than 0.01 were considered DEGs. EnhancedVolcano (81) was used for the data visualization. Functional enrichment of DEGs was performed using the hypergeometric test with the BH correction. Annotations were computed using the eggNOG-mapper with the eggNOG 4.5 orthology data (82). Pathway enrichment of DEGs was carried out by Pathview (83).

### EPC cell culture

To study the effect of *E. piscicida* on the cells, EPC cell line was used with minimum essential medium (MEM; Gibco, USA), 10% heat inactivated fetal bovine serum (FBS; Gibco, USA), 1% antibiotics-antimycotic (Gibco, USA), and 2.5% HEPES (Gibco, USA). Culture conditions were maintained using 60 mm cell culture dish (SPL, Republic of Korea) at 20°C. When the cultured cell lines were present in more than 90% of the field view, the adherent cells were detached by using 0.25% trypsin-EDTA (Gibco, USA) and seeded as 1 × 10^5^ cells in a new cell culture dish. The cells were sub-cultured at 3-day intervals, and cells with four or more passages were used for the experiment.

### Cell internalization and invasion assay

Internalization assay was adopted by the method described previously (84). To measure the internalization of *E. piscicida* to cells, epithelioma papulosum cyprini (EPC) was grown for 24 h to 80% confluence in 24-well plate. EPC monolayers were washed with PBS and then infected with *E. piscicida* in MEM at an MOI of (100:1) for 1 h at 27°C. The monolayers were washed five times with pre-warmed PBS and lysed with 1% Triton X-100. The number of bacteria was quantified by plating a dilution series onto BHI plates. For the cell invasion assay, EPC cells were first seeded in a 24-well plate, and the EPC monolayer was washed with PBS, followed by infection with *E. piscicida* in MEM at an MOI of (100:1) for 1 h at 27°C. After 1 h of incubation, monolayers were washed with pre-warmed PBS five times. Gentamicin was added to inhibit the growth of *E. piscicida* on the surface of the EPC cells, and the cells were lysed with 1% Triton X-100. Bacterial load was enumerated by plating the lysate on BHI plates.

### Statistical analysis

The significance of the differences between the mean values of the groups was initially validated, and the normality for all data was assessed by using the Shapiro-Wilk normality test. Multiple-comparison analysis was performed by using one-way analysis of variance (ANOVA) followed by the Brown-Forsythe test. If the data did not pass the normality test, a multiple-comparison analysis was performed by using the Kruskal-Wallis test followed by the Dunn test. *P* values of ≤0.05 were considered significant.

## Data Availability

The data sets analyzed during the current study are available in the Sequence Read Archive (SRA) under the number of SRP259317.

## AFTER EPUB

*[This article was published on 27 April 2026 with an error in the affiliations. The affiliations were corrected in the current version, posted on 1 June 2026.]*

## SUPPLEMENTAL MATERIAL

Supplemental material spectrum.00366-26-s0001.pdf Fig. S1 and S2; Tables S3 to S5.

Table S1 spectrum.00366-26-s0002.xlsx RNA-seq data set.

Table S2 spectrum.00366-26-s0003.xlsx Functional analysis data set.

ASM does not own the copyrights to Supplemental Material that may be linked to, or accessed through, an article. The authors have granted ASM a non-exclusive, world-wide license to publish the Supplemental Material files. Please contact the corresponding author directly for reuse.
