# Supplementary material for: Alteration of fimbria-mediated biofilm formation and virulence in the zoonotic pathogen Edwardsiella piscicida by sub-inhibitory concentrations of erythromycin exposures
Source: Microbiol Spectr. 2026 Apr 27;14(7):e00366-26. doi: 10.1128/spectrum.00366-26 (PMC13340299; doi:10.1128/spectrum.00366-26)
Supplement: Supplemental material — Fig. S1 and S2; Tables S3 to S5. [file spectrum.00366-26-s0001.pdf]

Figure S1.

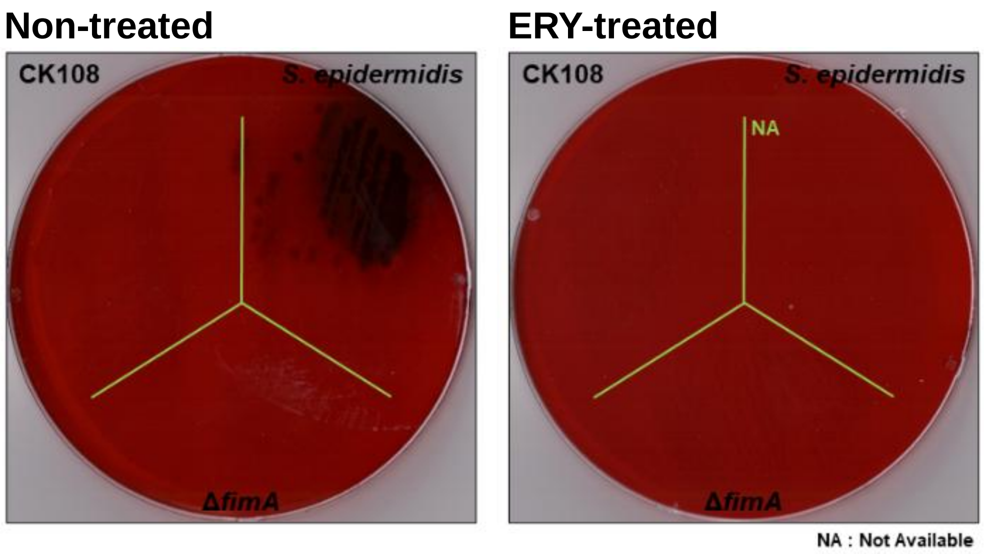

Supplementary Figure 1. Congo red assay.

E. piscicida streaks in BHI medium with congo red supplemented with 5% sucrose, grown in absence (-) or in presence (+) of sub-IC erythromycin. The plates were incubated for 48 hours at 27℃. Using the positive control as *Staphylococcus epidermidis*. Black-colored colonies indicate exopolysaccharide production.

Figure S2.

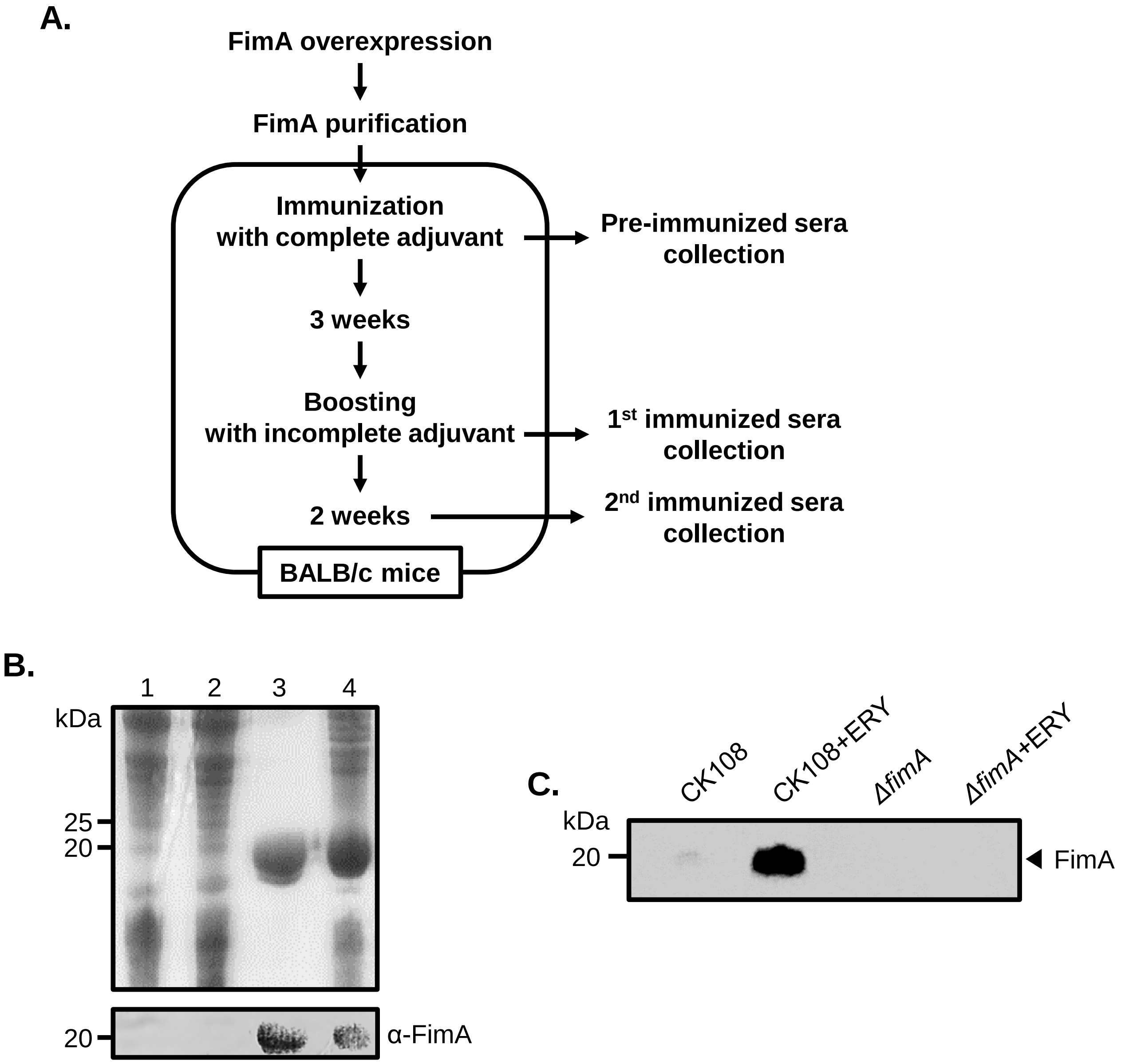

Supplementary Figure 2. Generation of FimA specific polyclonal antibody.

(A) Flow chart for processes of FimA specific polyclonal antibody production. (B) Confirmation of the produced FimA polyclonal antibody. In order to confirm the production of FimA-specific antibody, bacterial cell lysates and purified recombinant FimA protein were separated by SDS-PAGE analysis on 12% polyacrylamide gel (top). Protein bands were visualized by Simply blue^{TM} safe stain solution (Invitrogen). After SDS-PAGE analysis, immunoblot was performed with mouse sera (obtained from the 2nd immunization of FimA) diluted to the 1:1,000 ratio (v:v) (bottom). Lanes: 1, *Edwardsiella piscicida* CK108 total cell lysate; 2, *E. piscicida* CK108 Δ*fimA* total cell lysate; 3, Purified recombinant FimA protein; 4, FimA overexpressed *Escherichia coli* BL21(DE3)/pP$_{RO}$EX^{TM}HTb::*fimA*$_{CK108}$ total cell lysate. (C) Confirmatin of the anti-FimA antibody specificity and detection titer. Immunoblot analysis was performed with the mouse anti-FimA antibody diluted 1:1,000. Black arrow head indicate the immunoreactive protein.

Table S3. Bacterial strains used in this study

Strain	Characteristic	Reference
Escherichia coli		
DH5α	Transformation host for cloning	Invitrogen
BL21(DE3)	Transformation host for cloning and overexpression	Promega
χ7213	*E. coli* DH5α derivative (Δ*asd*), Km^R, DAP required	Sambrook & Russell, 2001
Edwardsiella piscicida		
CK108	Wild-type *E. piscicida* CK41 derivative, pCK41 cured	Yu *et al*., 2012
CK248	CK108 derivative, Δ*fap*Δ*fdp*	Choe *et al*., 2022
Δ*fimA*	CK108 derivative, Δ*fimA*	This study
Staphylococcus epidermidis		
BF14	Pathogenic wild-type	Lab. collection

Km^R; kanamycin resistance, DAP; diaminopimelic acid

Table S4. Plasmids used in this study

Plasmid	Characteristic	Reference
pGEM-T easy	Cloning vector for PCR product, ColE1 *ori*, Amp^R	Promega
$pP_{RO}EX^{TM}HTb$	Overrexpression vector, $LacI^q$, ColE1 *ori*, Amp^R	Invitrogen
pRE112	Suicide vector, R6K *ori*, *sacB*, Cm^R	Edwards *et al*., 1998
pWsk29	Cloning vector, pSC101 *ori*, Amp^R	Clements *et al*., 2002
pMM19	0.5 kb *fimA* DNA in pGEM-T easy vector, Amp^R	This study
pMM20	His6 fused 0.5 kb *fimA* DNA in $pP_{RO}EX^{TM}HTb$ vector, Amp^R	This study
pMM22	1.0 kb 3'-flanking DNA of *fimA* in pGEM-T easy vector, Amp^R	This study
pMM24	1.0 kb 5'-flanking DNA of *fimA* in pGEM-T easy vector, Amp^R	This study
pMM25	2.0 kb DNA containing 5'-flanking and 3'-flanking resions of *fimA* in pGEM-T easy vector, Amp^R	This study
pMM26	Derivative of pRE112, recombinant suicide plasmid for Δ*fimA*, *sacB*, Cm^R	This study
pMM27	0.5 kb *fimA* DNA in pWsk29, Amp^R	This study

Amp^R; ampicillin resistance, Cm; chloramphenicol resistance

Table S5. Primers used in this study

Primer	Nucleotide sequence*	Characteristic
KH108F_*Kpn*I	5'- ggtaccatcatgaacaccaac -3'	Forward for 5'-flanking of fimA
KH109R_*Bam*HI	5'- ggatcctatcataactccgtttat -3'	Reverse for 5'-flanking of fimA
KH100F_*Bam*HI	5'- ggatccttattcgtcgga -3'	Forward for 3'-flanking of fimA
KH101R_*Sac*I	5'- gagctcgactttgttgtc -3'	Reverse for 3'-flanking of fimA
KH102F_*Bam*HI	5'- ggatccaagaaaattttact -3'	Forward for fimA
KH103R_*Hin*dIII	5'- aagctttatttatattcga -3'	Reverse for fimA
KH118F	5'- ccctaagcggaatacccctt -3'	Forward for fimA confirmation
KH119R	5'- cgtcgttgatggccagcaat -3'	Reverse for fimA confirmation
KH80F	5'- gcctatgaaatcctgacc -3'	Forward for dnaJof qRT-PCR
KH81R	5'- gaagatatcgccgaacac -3'	Reverse for dnaJof qRT-PCR
KH121F	5'- agctaacggtaaagttgagt -3'	Forward for fimAof qRT-PCR
KH122R	5'- tatcgccaaccgctttaaat -3'	Reverse for fimAof qRT-PCR
KH153F	5'- ggccgatgtgatcgtgacta -3'	Forward for flhBof qRT-PCR
KH154R	5'- accttaggcgcactcatctt -3'	Reverse for flhBof qRT-PCR

*Underlines indicate restriction enzyme sites for the enzymes indicated in parentheses of the primer.
